# Titanium Elastic Nail Fixation Versus Spica Cast Application for the Treatment of Diaphyseal Femoral Fractures in Children Under Five Years Old: A Retrospective Study

**DOI:** 10.7759/cureus.77422

**Published:** 2025-01-14

**Authors:** Michael Zaidman, Naum Simanovsky, Vladimir Goldman, Reem Saleem-Zedan, Taer Abu Jabal, Eden Weisstub

**Affiliations:** 1 Department of Orthopedic Surgery, Hadassah Hebrew University Medical Center, Jerusalem, ISR

**Keywords:** femoral shaft fracture, outcome, pediatric population, spica cast, titanium elastic nails

## Abstract

Background

Femoral fractures are the most common type of injury requiring hospitalization in children. Treatment for femoral shaft fractures (FSFs) in children under six years old using closed reduction and spica cast (SC) application is a widely accepted method. In our institution, we offer the option of titanium elastic nail (TEN) fixation to parents of children under five years old. This study aimed to compare the results of SC treatment and TEN fixation in children under five years of age diagnosed with FSFs.

Methodology

We retrospectively reviewed medical records of all children under the age of five who had FSF treated at our institution and were managed with either an SC (28 children) or TEN fixation (26 children) between 2016 and 2022. The parents made the choice of treatment following discussions with the surgical team. In our analysis, the primary outcomes focused on radiological fracture healing and alignment, time until ambulation and limb mobilization, and complications. Additionally, we compared epidemiological data, hospitalization course, caregiver satisfaction, and follow-up duration between the two groups.

Results

No statistically significant differences were observed in time until fracture management or duration of anesthesia between the two groups. However, in the TEN group, short-term clinical and radiological outcomes were superior (coronal angulation: 0.92° vs. 5.9°, p = 0.0018; sagittal angulation: 0.42° vs. 7.82°, p = 0.0005; femoral shortening: 0.12 vs. 1.87 cm, p < 0.0001), although the time until ambulation was longer (5.9 vs. 4.75 weeks, p < 0.0001). Caregiver satisfaction was also higher in this group (p < 0.005). Conversely, children treated with TEN fixation more frequently required strong analgesic prescriptions (90% vs. 42%, p = 0.01), underwent more radiographs (during surgery: 6.73 vs. 3.61, p < 0.0001; during follow-up- 6.42 vs. 4.3, p = 0.003), had longer-lasting follow-up (7.9 vs. 3.2 weeks, p = 0.003), had an extended hospitalization (1.8 vs. 1.2 days, p = 0.004), and needed additional procedures for TEN removal. Both methods exhibited a low complication rate.

Conclusions

Titanium elastic nailing can be considered a viable treatment option for FSF in children aged two to five years, offering favorable clinical and radiological outcomes, enhanced caregiver satisfaction, and a low incidence of complications.

## Introduction

Femoral shaft fractures (FSFs), constituting approximately 2% of fractures in the pediatric population, rank among the most frequent limb fractures in children necessitating hospitalization [1-5]. This injury imposes a significant burden on both the patient and their family, potentially resulting in extended hospital stays, cast immobilization, disability, school absenteeism, and challenges in daily activities [1]. Moreover, it carries the risk of future complications, such as pain, skeletal asymmetry, and psychological effects [1,3]. The incidence of FSFs follows a bimodal pattern, with various injury mechanisms associated with different age groups. These include child abuse, minor trauma, and pathologic fractures in early childhood, while road traffic accidents, falls from height, sports-related injuries, and incidents involving heavy objects impacting the affected limb are more common in older children [1,3].

Several treatment options are available for pediatric FSFs, including skeletal or skin traction, hip spica cast (SC) immobilization, closed reduction with minimally invasive plate osteosynthesis, external fixator application, and internal fixation using titanium elastic nails (TENs) [2,3]. The choice of treatment strategy depends on various factors such as the patient’s age, presence of other injuries, comorbidities, the pattern and properties of the fracture, as well as socioeconomic considerations [2]. When treating femoral fractures in children, the decision regarding the treatment method is typically influenced more by the surgeon’s personal preference and the family’s considerations. This is because pediatric femoral fractures generally heal well over time due to the natural bone remodeling process, making the specific type of treatment less critical to the healing outcome [6]. Many surgeons prefer elastic, stable intramedullary nailing using TENs for treating long bone fractures in children. This preference is attributed to the technique’s minimally invasive nature, which offers significant advantages, such as reduced tissue trauma, shorter recovery times, and a lower risk of postoperative complications [7-11]. Typically, children under the age of four with FSF are treated conservatively using a hip SC, which enables early discharge from the hospital [12,13]. However, it is important to note that managing treatment with an SC can be demanding [14].

In a study conducted by Flynn et al., it was observed that patients with FSF aged 6-16 years experienced significantly faster recovery and fewer complications when treated with TENs compared to those managed with traction and SC [15]. This finding was validated by Saseendar et al., who conducted a similar study in children aged 5-15 years with FSF [16]. They reported that TEN fixation resulted in better outcomes, including earlier union and lower rates of malunion [16]. While there is a consensus on the treatment approach for infants and adolescents, the optimal management strategy for toddlers and preschool-aged children below the age of five to six years remains a subject of debate [1,5,17-21]. To emphasize the difference in approaches, one can compare the clinical guidelines provided by the American Academy of Orthopedic Surgery, which recommends traction with SC application for children between the ages of six months and five years, with the recommendations of the German Society of Pediatric Surgery, which suggests the use of flexible intramedullary nails for treating FSF in children older than three years [22].

This study aimed to assess and compare the outcomes of TEN and SC treatments for FSFs in children under the age of five years.

## Materials and methods

The study was approved by the Institutional Review Board of Hadassah Hebrew University (approval number: 0702-18 HMO; 27/12/2018). We conducted a retrospective review of medical records and radiographs for children under the age of five years with FSF who were treated at our institution between January 2016 and December 2022. Children who underwent closed reduction and the application of an SC, as well as those treated with TEN fixation (PediFlex™, OrthoPediatrics), were included in the study and categorized into their respective groups. Children over five years old, cases where the SC was replaced with TEN, and those who received additional external fixation augmentation for TEN fixation were excluded from the study. The choice of treatment was determined through discussions between the parents and the surgical team. The skin traction was routinely applied in the emergency room to maintain pain control and prevent femoral shortening until the definite treatment. The epidemiological data encompassed age, weight, gender, relevant medical history, the side of the fracture, concomitant injuries, and cases of polytrauma. Hospitalization data included the time from admission to definitive fracture management, duration of anesthesia, need for opioid analgesic prescriptions during hospitalization, and length of the hospital stay. Clinical and radiological outcomes, as well as follow-up analysis, included the time until ambulation, the degree of coronal and sagittal angulation, and fracture shortening, which were measured during outpatient visits when ambulation was allowed. Additionally, we assessed complications, the number of X-rays performed, and the number of follow-up outpatient visits. Overall caregiver satisfaction was evaluated using Single Assessment Numeric Evaluation. During the follow-up visits, parents were asked to rate the difficulty of caring for their child on a scale from 0 to 100, with 0 indicating no difficulty and 100 representing the most challenging scenario imaginable. Responses below 50 were considered reasonable and not overly challenging.

In our study, we focused on primary outcomes, including radiological fracture alignment and healing, time until ambulation, and complications. We compared these primary outcomes between the two groups, alongside examining secondary outcomes such as differences in the hospitalization course, caregiver satisfaction, and follow-up duration.

All procedures were performed by one of four staff pediatric orthopedic specialists from our department, with assistance either from another pediatric orthopedic specialist or an orthopedic resident.

Spica cast application (the authors’ preferred technique)

**Figure 1:**
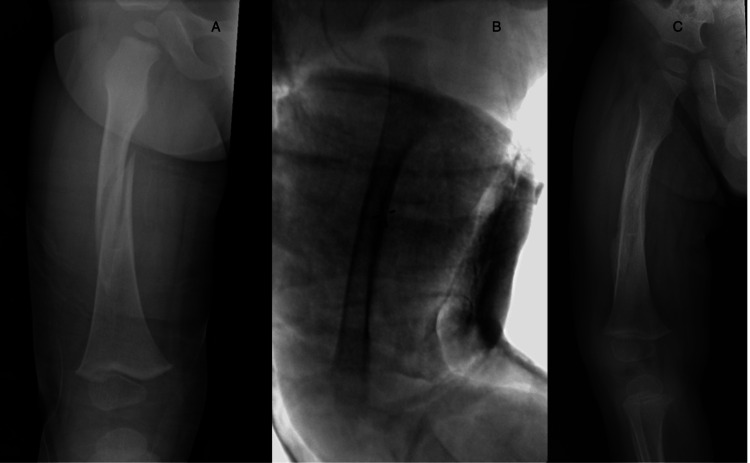
Original perioperative images of our spica cast treatment. A 19-month-old boy with a right femoral shaft fracture (A). Intraoperative fluoroscopy image showing reduction and spica cast immobilization (B). Follow-up image showing a good radiological result one month after surgery (C).

In our institution, the application of the hip SC is done within an operating room setting under general anesthesia. While a basic technique has been described by Tisherman et al. [23], we propose a modified technique that leads to improved fracture reduction and alignment (Figure 1).

The procedure involves positioning the patient on a hip spica table, with the recommendation for the involvement of at least two surgeons. Initially, an above-knee plaster of Paris (POP) cast is applied to the fractured extremity with the knees bent at 45-70°, allowing it to harden. This modification aids in subsequent fixation, fracture reduction, maintaining traction during cast hardening, and preventing femoral shortening. It is crucial to use an above-knee POP cast to prevent undue pressure on the neurovascular structures of the popliteal fossa, which can occur if the edges of a below-knee cast harden after knee flexion. To ensure the child’s comfort and unrestricted breathing, a folded towel is placed between the abdominal wall and the padding-cast layer. We advise starting with a relatively thin layer of POP to achieve optimal molding, followed by the application of a fiberglass cast for reinforcement. We wrap both legs with fiberglass cast tape, forming an elliptical shape. Additional fiberglass tape is then applied between the legs to construct a supportive “bar” that connects them. This reinforcement strengthens the cast fixation, ensuring added support and stability, resulting in a well-molded and sturdy SC. Another important aspect is molding the cast at the fracture site with a slight valgus alignment, as most midshaft fractures tend to further shift into varus. It is crucial to confirm fracture alignment after the reduction maneuver and to verify the maintenance of reduction after applying the SC using a C-arm intensifier. For enhanced stability, we prefer a one-and-a-half SC. Moreover, it is essential to ensure that the SC provides adequate access to the perineal area. Patients treated with an SC are scheduled for a follow-up examination four to five weeks after cast application. At this appointment, the cast is removed, and ambulation is permitted if X-ray images confirm fracture healing. A second follow-up is then arranged for six weeks later, and if all is well, subsequent follow-ups are scheduled as needed.

Titanium elastic nail fixation operative technique

**Figure 2:**
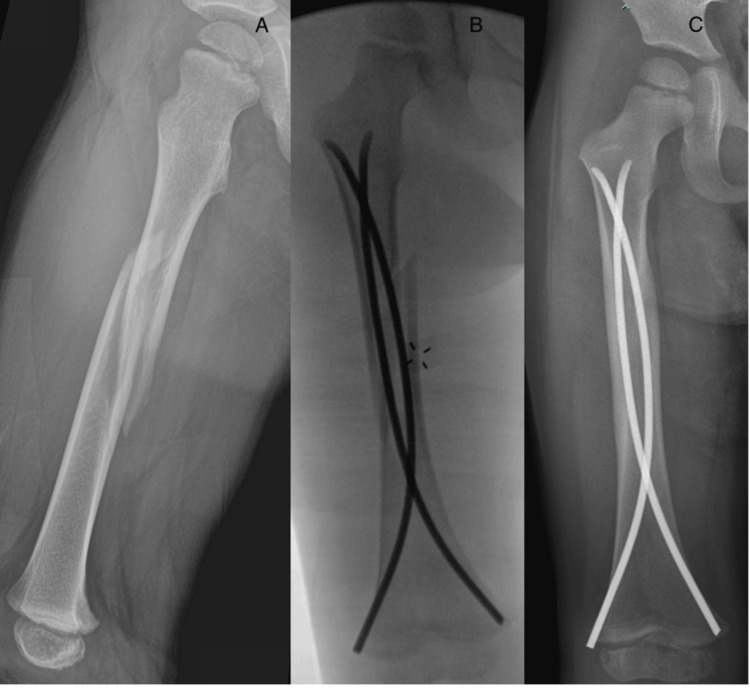
Original perioperative images of titanium elastic nail fixation. A 3.5-year-old boy with a right femoral shaft fracture (A). Intraoperative image showing the fracture fixation using two titanium elastic nails (B). An X-ray image taken four months after surgery showing a well-aligned femoral shaft (C).

Upon admission to the emergency room, we initiate skin traction equivalent to 10% of the child’s body weight. This measure serves to prevent excessive fracture shortening and provides effective pain relief. For the insertion of TENs, we calculate their diameters as 40% of the narrowest diameter of the femoral intramedullary canal, as measured on both the anterior-posterior (AP) and lateral radiographs. The patient is positioned on a radiolucent table, with a bolster placed under the foreleg of the fractured extremity and a folded towel positioned beneath the unilateral sacral area to elevate it over the contralateral leg. This setup allows us to obtain AP and lateral C-arm images by rotating the C-arm intensifier, confirming the reduction in both planes without the need to move the leg. It is worth noting that lifting the leg for lateral view visualization after achieving alignment on the AP view can often result in a loss of alignment. Two retrograde TENs are inserted through the distal femoral metaphysis. To achieve optimal fracture stability, the nails should be identically pre-bent with their apex positioned at the level of the fracture. In young children, where the distal part of the metaphysis may be intra-articular, the bone perforation for nail insertion should begin slightly proximal compared to older patients (Figure 2).

In cases where the proximal diaphysis is displaced laterally, inserting the lateral retrograde nail first can help reduce the fracture and facilitate the medial nail insertion. Conversely, if the medial nail is inserted first, it may worsen lateral translation, making the lateral nail insertion more challenging or even impossible. The opposite order is recommended when the proximal diaphysis is displaced medially. In situations where fracture shortening is significant, the anesthesiologist may prescribe short-term relaxants to ease fracture reduction. A soft dressing is applied at the end of the procedure. We do not apply a cast after fracture fixation with TENs, which allows for immediate limb mobilization and the beginning of early (first postoperative day) physical therapy treatment. Patients who undergo TEN fixation are called for their first follow-up five to six weeks postoperatively, followed by another visit six weeks later. The TENs are typically removed six to eight months post-surgery.

Statistical analysis

Data from both groups were calculated and compared, and a statistical analysis was conducted. The Student’s t-test was applied to compare quantitative variables, while the Shapiro-Wilk test was used to assess the normality of data distribution. In instances of non-normal distribution, the Mann-Whitney U test was used. For categorical variables, the chi-square test was employed. Statistical analyses were performed using SPSS Statistics for Windows, Version 26.0, released in 2019 (IBM Corp., Armonk, NY, USA).

## Results

This study included 54 patients. Of these, 26 (48.15%) children underwent TEN fracture fixation, while 28 (51.85%) were treated using closed reduction and SC application.

Epidemiological data

The mean age in the TEN fixation group was 3.68 years, ranging from two to five years. The average weight was 16.29 kg, ranging between 12 and 20 kg (Table 1).

**Table 1:** Demographics of the study population. *: Statistically significant p-value using Student’s t-test (p < 0.05); **: chi-square test. SD: standard deviation

Variables	Spica cast (n = 28)	Titanium elastic nails (n = 26)	P-value
Mean age (years)	1.96 (SD = 0.483)	3.68 (SD = 0.756)	<0.01*
Mean weight (kg)	11.84 (SD = 1.95)	16.29 (SD = 2.826)	<0.01*
Gender (male, %)	23 (82%)	19 (73%)	>0.5**
Side of injury (right, %)	14 (50%)	11 (46%)	>0.5**
Polytrauma (%)	2 (7%)	3 (11.5%)	>0.5**
Open fracture (%)	0 (0%)	1 (4%)	>0.5**
Comorbidities (%)	1 (3.5%)	0 (0%)	>0.5**

In contrast, the average age in the SC fixation group was 1.96 years, with a range of one to five years (p < 0.0001). The average weight was 11.84 kg, with weights ranging from 7.5 to 19 kg (p < 0.0001). There were no significant differences between the groups regarding gender, side of injury, and prevalence of polytrauma. The composition of the SC group included a predominance of males, accounting for 23 (82%) children, while the TEN group also had a majority of male participants, comprising 19 (73%) children. In the TEN fixation group, 11 (46%) had fractures on the right side of the femur compared to 14 (50%) in the SC fixation group. Polytrauma, encompassing injuries to the head, abdomen, thorax, or spine, was observed in three (11.5%) children in the TEN group and two (7%) children in the SC group. One patient in the SC group had osteogenesis imperfecta. One patient in the TEN group sustained an open femoral fracture.

Hospitalization course

The mean duration from admission to definitive fracture management was 27.6 hours (ranging from two to 40 hours) for the TEN group and 16.9 hours (ranging from three to 47 hours) for the SC group, with no significant difference (p = 0.12) (Table 2).

**Table 2:** Hospitalization course. *: Statistically significant p-value using Student’s t-test (p < 0.05); **: Mann-Whitney test. SD: standard deviation

Variables	Spica cast	Titanium elastic nails	P-value
Duration until treatment (hours)	16.04 (range = 3-47)	27.62 (range = 2-40)	0.12**
Duration of anesthesia (minutes)	43 (SD = 14.9)	50.65 (SD = 13.7)	0.06
Length of hospital stay (days)	1.2 (SD = 0.5)	1.8 (SD = 0.59)	<0.01*

It is important to note that the initiation of definitive treatment is not solely dependent on orthopedic management but is primarily influenced by the patient’s overall health condition, assessments by other medical specialties (such as emergency medicine, pediatrics, etc.), and the treatment of any additional injuries. Anesthesia duration was also comparable between the two groups at 43 minutes for the SC group and 50.65 minutes for the TEN group (p = 0.06). The average length of hospital stay was slightly longer for the TEN group at 1.8 days compared to 1.2 days for the SC group, with a statistical significance (p = 0.004). Patients in the TEN group were prescribed strong analgesics (Oxycode) more than twice as frequently as those in the SC group, with rates of 90% compared to 42% (p = 0.012). Both treatment methods demonstrated a low complication rate during the postoperative period. Within the SC cohort, complications were observed in 7.14% of the cases, including one individual developing a pressure ulcer attributable to the cast, while another experiencing cast failure, mandating reapplication under general anesthesia. In the TEN cohort, a complication rate of 3.84% was noted, with one instance of nail protrusion secondary to fracture shortening, necessitating surgical nail adjustment in an operation room setting.

Clinical and radiological outcomes

The initiation of ambulation post-treatment was notably quicker in the spica fixation cohort, averaging 4.75 weeks, compared to the 5.9 weeks observed in the TEN fixation cohort (p < 0.0001) (Table 3).

**Table 3:** Clinical and radiological outcomes. *: Statistically significant p-value using Student’s t-test (p < 0.05). SD: standard deviation

Variables	Spica cast	Titanium elastic nails	P-value
Initiation of ambulation (weeks)	4.75 (SD = 0.83)	5.9 (SD = 0.91)	<0.01*
Coronal angulation (°)	5.9 (SD = 7.51)	0.92 (SD = 1.72)	0.01*
Sagittal angulation (°)	7.82 (SD = 10.0)	0.42 (SD = 1.27)	<0.01*
Femoral shortening (cm)	1.87 (SD = 0.715)	0.12 (SD = 0.2)	<0.01*

Upon assessment during outpatient visits after ambulation was permitted, the TEN fixation cohort exhibited superior outcomes in parameters of coronal and sagittal angulation, as well as femoral shortening. The TEN cohort had a coronal angulation average of 0.92° (range = 0°-6°) compared to 5.9° (range = 0°-27°) in the spica cohort (p = 0.002). Sagittal angulation averaged 0.42° (range = 0°-4°) in the TEN group and 7.82° (range = 0°-42°) in the spica group (p < 0.0005). Femoral shortening was 0.12 cm (range = 0-0.6 cm) in the TEN group and 1.87 cm (range = 0-3.4 cm) in the spica group (p < 0.0001).

Caregiver’s satisfaction

All parents mentioned the challenge of caring for their children during the SC treatment phase (mean score = 85). In contrast, parents of children treated with nail fixation reported no such difficulties (mean score = 36.2). This difference demonstrated statistical significance with a p-value smaller than 0.005. The distinction was particularly felt by caregivers in cases where an SC was converted to nail fixation.

Follow-up and number of radiographs obtained

The TEN fixation group was characterized by a longer follow-up period, averaging 7.9 months (range = 3.5-20 months), compared to the SC group’s follow-up period of 3.2 months (range = 1-11 months) (p = 0.03) (Table 4).

**Table 4:** Follow-up and number of radiographs obtained. *: Statistically significant p-value using Student’s t-test (p < 0.05). SD: standard deviation

Variables	Spica cast	Titanium elastic nails	P-value
Follow-up period (months)	3.2 (SD = 7.27)	7.9 (SD = 3.82)	0.03*
Outpatient visits	2.3 (SD = 0.99)	3.58 (SD = 1.17)	<0.01*
Number of radiographs in the emergency department	2.43 (SD = 0.88)	2.73 (SD = 1.04)	0.26
Number of radiographs during surgery	3.61 (SD = 2.04)	6.73 (SD = 2.22)	<0.01*
Number of radiographs during follow-up	4.3 (SD = 2.48)	6.42 (SD = 2.52)	<0.01*

On average, the TEN group had 3.58 outpatient visits, while the SC group had 2.3 (p < 0.001) Children undergoing nail fixation not only had more radiographs during the procedure, 6.73 compared to 3.61 (p < 0.0001), but also during the follow-up visits, 6.42 versus 4.3 (p = 0.003). There was no significant difference in the number of radiographs taken in the emergency room at 2.73 for TEN versus 2.43 for SC (p = 0.26). In our institution, hardware removal is standard practice. Consequently, all patients in the TEN group underwent nail removal, on average, 8.5 months after fracture fixation.

## Discussion

In trauma surgery, the goal is to restore function and expedite the patient’s return to their pre-injury activity level. This study was designed to evaluate the outcomes of SC treatment versus TEN fixation in children under five years of age who had sustained FSF. Our primary endpoints included assessment of radiological fracture healing and alignment, duration until ambulatory capability and limb mobilization are regained, and the incidence of complications. Regarding short-term clinical and radiological outcomes, the TEN group demonstrated an advantage.

In line with the approach described by Pogorelic et al. and practiced in our institution, we do not apply a cast following TEN fixation, enabling immediate limb mobilization [11]. TEN fixation allows for immediate extremity mobilization and maintains better fracture alignment until bridging callus formation occurs [22]. This accelerates the rehabilitation process and minimizes muscle contractures by enabling an earlier return to activity. In our study, it was also observed that the SC group was allowed to bear weight on the injured leg earlier than the TEN group. We hypothesize that the variation in the onset of ambulation can be attributed to the age discrepancy among the groups. Specifically, children around two years old are permitted to begin walking earlier due to quicker callus formation and reduced risk of fracture displacement. Li et al. provided evidence suggesting that patients weighing over 40 kg who undergo stabilization of a transverse midshaft femur fracture with TENs are at risk of reduction loss in both sagittal and coronal planes [24]. Consequently, it might be advisable to consider allowing children under the age of four to bear weight on their operated leg earlier.

Both techniques exhibited a low complication rate. In line with the findings presented in the research conducted by Wall et al., our study also observed a comparable rate of implant revision, approximately 5% [8]. In the TEN cohort, no patient experienced clinically significant shortening, but one patient required a revision due to distal nail protrusion. Complications associated with SC included dermatological issues attributed to inadequate hygiene, cast breakage, and fracture shortenings exceeding 2 cm.

One of the most significant factors influencing parental choice of treatment is the difficulty in caring for a child in a long-term SC. This challenge becomes particularly noticeable when transitioning from SC to TEN fixation. Children receiving treatment with an SC require continuous assistance with mobility and transportation, as well as support with toileting, often necessitating home care [14]. However, TEN fixation also has its disadvantages, as highlighted by our study findings. These drawbacks include a heightened need for potent analgesics, an increased number of radiographic images taken during the procedure, an extended hospital stay, and longer follow-up times. Additionally, the need for an extra procedure to remove the TEN implants prolongs the follow-up duration and mandates additional radiographs.

Both SC and TEN represent viable treatment modalities for FSF in the pediatric population. Both methods entail significant social, economic, educational, and emotional costs [1,2,14,17,22,23,25]. The choice between these approaches often hinges on the surgeon’s experience and personal preference. At our institution, we empower parents to select the treatment approach following comprehensive discussions and explanations of both options. While both methods have their advantages and disadvantages, neither unequivocally surpasses the other. Therefore, we stress the importance of granting parents the authority to make the final decision. Traditionally, the majority of surgeons treating pediatric FSF opt for conservative management when dealing with children under the age of five years [22,26-28]. However, recent trends indicate a notable shift, with an increasing number of children, particularly those between the ages of four to six years, now being treated with TEN instead of the previously more common SC for diaphyseal femur fractures [28]. Our study demonstrates that TEN fixation is an effective treatment for patients with an average age of around 3.5 years. The operative technique outlined simplifies the application of TEN for FSF fixation in toddlers, starting from the age of two years. This approach shows promise for this specific age group and has the potential for broader implementation in clinical practice.

The limitations of this study include its retrospective design, limited sample size, variations in patient age and weight, and the absence of long-term follow-up. Future studies should, therefore, aim for larger cohorts with long-term follow-up and consider a prospective approach, with an emphasis on stratifying patients by age and weight to gain more detailed insights.

## Conclusions

TEN can be considered a valid option for treating FSF in children aged two to five years, providing good clinical and radiological results and enhanced caregiver satisfaction with a low complication rate. In polytrauma patients with thoracic-abdominal injuries or open fractures, the use of TEN may be favored. This is particularly the case when the use of an SC is impractical or inadvisable. Further research involving a larger patient population and a prospective study design is necessary to comprehensively evaluate the effectiveness of TEN in treating pediatric patients under the age of five years.
